# From Donor to the Lab: A Fascinating Journey of Primary Cell Lines

**DOI:** 10.3389/fcell.2021.711381

**Published:** 2021-07-22

**Authors:** Magdalena Richter, Oliwia Piwocka, Marika Musielak, Igor Piotrowski, Wiktoria M. Suchorska, Tomasz Trzeciak

**Affiliations:** ^1^Department of Orthopaedics and Traumatology, Poznan University of Medical Sciences, Poznań, Poland; ^2^Radiobiology Lab, Department of Medical Physics, Greater Poland Cancer Center, Poznań, Poland; ^3^Department of Electroradiology, Poznan University of Medical Sciences, Poznań, Poland

**Keywords:** primary cancer cell lines, tumour dissociation, isolation of the cell lines, 2D versus 3D cell culture, methods for cell line characterisation

## Abstract

Primary cancer cell lines are *ex vivo* cell cultures originating from resected tissues during biopsies and surgeries. Primary cell cultures are objects of intense research due to their high impact on molecular biology and oncology advancement. Initially, the patient-derived specimen must be subjected to dissociation and isolation. Techniques for tumour dissociation are usually reliant on the organisation of connecting tissue. The most common methods include enzymatic digestion (with collagenase, dispase, and DNase), chemical treatment (with ethylene diamine tetraacetic acid and ethylene glycol tetraacetic acid), or mechanical disaggregation to obtain a uniform cell population. Cells isolated from the tissue specimen are cultured as a monolayer or three-dimensional culture, in the form of multicellular spheroids, scaffold-based cultures (i.e., organoids), or matrix-embedded cultures. Every primary cell line must be characterised to identify its origin, purity, and significant features. The process of characterisation should include different assays utilising specific (extra- and intracellular) markers. The most frequently used approaches comprise immunohistochemistry, immunocytochemistry, western blot, flow cytometry, real-time polymerase chain reaction, karyotyping, confocal microscopy, and next-generation sequencing. The growing body of evidence indicates the validity of the usage of primary cancer cell lines in the formulation of novel anti-cancer treatments and their contribution to drug development.

## Introduction

**FIGURE 1:**
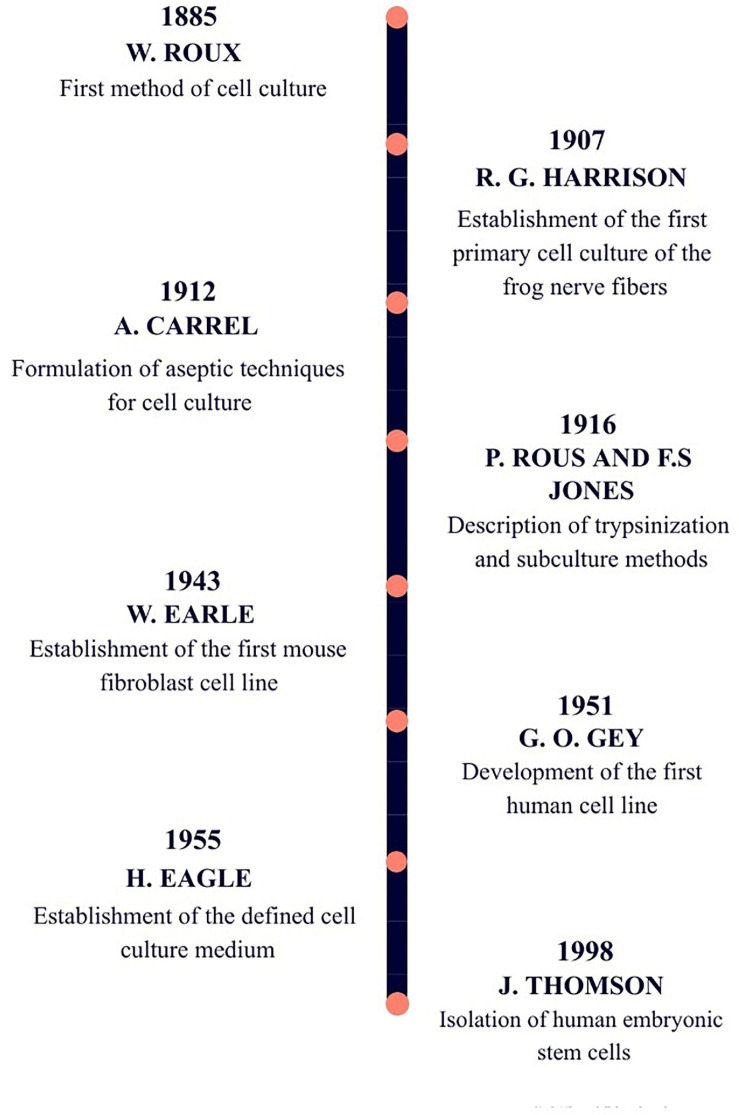
The timeline of key achievements that contributed to the development of cell culture.

Cell culture development significantly changed the area of life sciences and contributed to great advancements in medicine. Research with the use of cell lines is an essential procedure for modelling diseases, stem cell and cancer investigation, and the establishment of therapies (Jedrzejczak-Silicka, 2017). The first observations leading to the development of cell culture (Figure 1) were done in the 1800s by Wilhelm Roux, who maintained chicken embryos alive for few days in saline solution, thereby, proposing the principle of cell culture (Hudu et al., 2016). However, the exact primary cell culture has its beginning in the first decade of the 20th century, when Ross Granville Harrison successfully cultivated frog nerve cells by hanging drop method. For this experiment, he used small pieces of frog embryonic tissue immersed in droplets of lymph solution on the cover slide. Then, he turned a plate upside down and to great effect maintained a primary cell culture, in which he observed nerve cells and watched developing fibres (Jedrzejczak-Silicka, 2017). After these achievements, cell culture was improved by elaboration of work under aseptic conditions, which led to other discoveries, such as, the establishment of the first mouse fibroblast culture, followed by the development of the first human tumour cell line. A few years later, MEM and DMEM media were formulated, as well as media supplemented with growth factors. The last crucial event for the development of cell culture, took place in 1998 when human embryonic stem cells were isolated (Jedrzejczak-Silicka, 2017; Ledur et al., 2017).

Cell lines, as we know them today, play a remarkable role in cancer research. The application of cancer cell lines enabled a better understanding of tumour biology and improved drug development (Mitra et al., 2013). The examples of drugs formulated with the use of immortalised cell lines are trastuzumab tested on breast cancer cultures (Zazo et al., 2016), imatinib tested on myelogenous leukaemia cell line (Tusa et al., 2020) or bevacizumab used for therapy of glioblastoma (Simon et al., 2014) and non-small cell lung cancer (Lauro et al., 2014).

The main aim of this review is to present the most recent data referring to primary cell lines isolation and characterisation processes. This article highlights the variety of isolation methods depending on the cells’ source and its next implementation. Moreover, we aimed to introduce different molecular methods of isolated cells’ characterisation. This review is a summary of the available knowledge in the field of primary cell lines culture.

## Primary Versus Established Cell Lines

Primary cancer cell lines are *ex vivo* cell populations deriving directly from resected tissue samples, most commonly from core biopsies, fine-needle aspirates, pleural effusions, resections, or autopsy specimens (Kodack et al., 2017; Miserocchi et al., 2017). These samples can reflect the tumour’s natural microenvironment and preserve characteristic crosstalk between healthy and cancerous cells. Those intercellular behaviours are essential during carcinogenesis, progression, and metastases, and also take part in responses to therapies (Leithner et al., 2014; Miserocchi et al., 2017). Primary cultures preserve the stem-like phenotype of cancer cells, which is also valuable during preclinical studies of drug resistance mechanisms. *Ex vivo* models enable accurate reflection of tumours and are more suitable for clinical analysis, in contrast to the most widely used immortalised cell lines that may not be completely predictive towards cancer expression (Miserocchi et al., 2017). Genetic aberrations of immortalised cell lines accumulate with every passage, limiting their usefulness and impacting the final overview. There is also variation in patient reactions to the same drugs used on tumours with identical genetic mutations. It is complicated to comprise all genetic and epigenetic variants’ heterogeneity in millions of patients by having at disposal only a limited number of cancer cell lines. Hence, there is growing interest and need to establish primary cancer cell lines (Mitra et al., 2013).

Primary cancer cultures serve as a powerful tool for studying cancer biology, gene expression, oncogene activation, or amplification. Patient-derived specimens are useful in examining hormone responsiveness and chemotherapy’s impact on tumour cells (Vázquez et al., 2004). However, primary cells have a finite lifespan and limited replicative capacity (Table 1), which leads to narrow culture time (Gillooly et al., 2012). Depending on the goals of the research, scientists should choose the appropriate model to fit the needs of the experiment. The introduction of established cell lines has led to the development of many therapies and treatment strategies. Moreover, it has allowed to work on the same lines in different places around the world. This fact is a key role in comparing the achieved results, drawing conclusions, and producing better cancer treatment strategies. Nevertheless, the short-term culture of primary cells from solid tumours acquired great importance in personalised cancer therapy (Mitra et al., 2013). Combined with next-generation sequencing (NGS), primary cancer cultures create a promising cancer treatment tool (Ishikawa, 2017).

**TABLE 1:** Comparison of primary and immortalised cell lines.

**Feature**	**Primary cells**	**Immortalised cell lines**	**References**
Biological relevance	Higher	Lower	Mitra et al., 2013
Lifespan and proliferation	Limited	Unlimited	Gillooly et al., 2012
Molecular properties	Preserve characteristic cells’ behaviours and cross-talk between cells	Untypical cellular behaviours may occur	Carter and Shieh, 2010; Miserocchi et al., 2017
Phenotype	High heterogeneity	Lower heterogeneity	Ali et al., 2017
Genetics	Original genome	Altered genome, accumulation of genetic aberrations	Carter and Shieh, 2010
Relevance *in vivo*	Best experimental models for *in vivo* studies	Low relevance for *in vivo* studies	Verma et al., 2020
Handling	Needs optimised culture conditions and media	Established media from manufacturer	Weigand et al., 2016
Ethics	Require Ethical Commission approval and patient consent	Do not require additional documents	Petrini, 2012

## Steps Before Proceeding to Experiment

### Ethical and Legal Requirements in Europe

Human biological materials are valuable specimens, widely used for studying health-related issues. These components are unique for every human and are obtained from various body parts during the examination, surgery, or post-mortem. Biological materials comprise DNA, proteins, cells, tissues (including blood and plasma), whole organs, saliva, urine, faeces, and other body liquids (i.e., wound fluids). Those are gathered for different purposes: diagnosis, clinical treatment, or research (Patel and Rajput, 2019). Laboratories that possess and handle biological samples must consider numerous issues connected to bioethics, legislation, and storage. The research approach on human biological materials differs among people, thus researchers must respect and take donors’ morals under advisement (Petrini, 2012).

The Clinical Trials Regulation EU No 536/2014 (CTR) modulates research on biological materials of human origin. According to those directions, obtaining biological samples must be held only with the “donors” written informed consent. Alternatively, an authorisation may be given on behalf of the donor by an eligible person or representative authority. Written informed consent should enable the right to withdraw the approval and alter the scope of the agreement. The person concerned can stipulate the use of his/her biological material and personal data at any time. However, when gathering “patients” information, it is hard to foresee the purpose and scope of personal data processing for future research. Thus, it should be allowed to consent to certain areas of study, depending on the “donors” wishes. Alternatively, it is advised to compose an additional article of the consent, stating that the donor permits to use his/her data outside of the clinical trial protocol.

Research should commence only after an independent evaluation of its scientific value, including the significance of the aim and confirmation of ethical standards by the appropriate committee [Casali and Vyas, 2021, Regulation (EU) No 536/2014]^1^.

### Storage and Disposal of Human Biological Materials

Biospecimens are usually collected during surgery or examination (Patel and Rajput, 2019). For some studies, the samples must be appropriately preserved (Hubel et al., 2014). The most frequently used methods of preserving human biological material are freezing and storage at low temperatures or chemical fixation (Hubel et al., 2014). Biospecimens of human origin are treated as biohazardous and after used most commonly are disinfected and disposed of by incineration (Patel and Rajput, 2019).

### Preparation for the Experiment

Human biological samples must be treated as potentially dangerous materials since these might be contaminated with contagious pathogenic viruses (e.g., HIV or hepatitis viruses), methicillin-resistant *Staphylococcus aureus*, bacteria, and fungi (Shepherd et al., 2007; Geraghty et al., 2014). For these reasons, tissue processing should be executed aseptically in a Class II biosafety cabinet. Biospecimen collection shall be performed by a surgeon and pathologist (Yap et al., 2019). Authors of research should include histopathological diagnoses, confirmation of how the specimens were collected (biopsy or resection), description of the tumour and its origin (primary tumour site or metastatic lesion), as well as procedure applied to fixed tissue (formalin-fixed paraffin-embedded or frozen tumour tissue) (Nushtaeva et al., 2019). As far as possible, data on preanalytical handling of specimens should also be given. If control samples are used in research, their origin and isolation should also be noted. Besides describing the specimen, authors need to include patients’ characteristics, such as age, gender, ethnicity, medical history, or disease stage. Thus it might be relevant for research as in cohort studies (Knijn et al., 2015). Also, a small amount of non-cancerous tissue should be frozen to indicate other genetic differences of a cell line and can be used in the process of authentication (Geraghty et al., 2014).

## Methods of Tumour Dissociation and Isolation of the Primary Cell Lines From an Explant

### Tumour Dissociation

**FIGURE 2:**
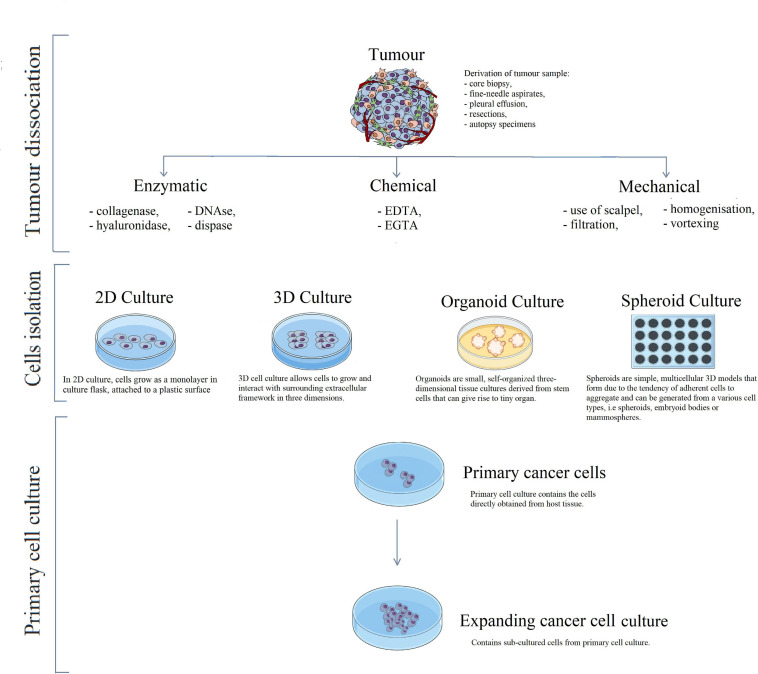
Schematic visualisation of primary cancer cell line development. Cancerous tissue can be dissociated by three different methods. The enzymatic method utilises enzymes such as collagenase, dispase, DNase, depending on tissue structure. The chemical method makes use of EDTA or EGTA that causes the loosening of intercellular bonds. Methods are often combined, for example mechanical tissue disaggregation with a scalpel, followed by enzymatic digestion. Afterwards, tumour tissue is seeded onto a plate, and cells are grown in 2D culture or 3D culture, in form of organoids or spheroids. The last step of primary cell line development is cell culture in appropriate conditions and further expansion of cancer culture.

Techniques for tumour dissociation are usually reliant on the organisation of connecting tissue. To obtain a uniform cell population, the most common methods include enzymatic digestion, chemical treatment, or mechanical dissociation (Figure 2; Mitra et al., 2013).

#### Enzymatic Digestion

The usage of enzymes for tissue dissociation is a general procedure that enables the retainment of cell viability and integrity by concurrent decent tissue digestion (Table 2). The most widely used enzyme is collagenase; however, it can be used with other enzymes (Mitra et al., 2013). Collagenase belongs to the class of endopeptidases and digests collagen in the region of the triple helix. Collagen is the leading component in connective tissue; thus, collagenase is useful when isolating epithelial, endothelial, or adrenal tissue. Moreover, it can be applied for the isolation of adipocytes, hepatocytes, cardiomyocytes, and the treatment of mammary or other soft tissues^2^. The procedure of cell isolation with collagenase and hyaluronidase is also successful in breast cancer (Pandrangi et al., 2014; Zubeldia-Plazaola et al., 2015; Weigand et al., 2016). There are different approaches concerning the mixture of those two enzymes, for example, slow/fast digestion shown by Zubeldia-Plazaola et al. (2015). Their research tested the efficacy of using collagenase and hyaluronidase of different concentrations for various periods. During slow digestion, human tissue was left overnight at a low enzymatic concentration of collagenase and hyaluronidase, 1.6 and 0.14 mg/mL, respectively. Fast digestion lasted for 4–6 h, and the concentration of used collagenase and hyaluronidase was equal to 2 mg/ml, in both cases. Slow and fast digestion was then followed by sequential filtering and differential centrifugation that resulted in organoids, epithelial, and stromal fractions. This study indicates the validity of slow digestion due to the more significant number of obtained viable cells that gave rise to a better quantity of cells in culture than fast digestion. Slow digestion is a less aggressive method, thus, it is possible to acquire higher cell yield. Moreover, slow digestion followed by differential centrifugation enables more significant cell growth and efficacy than sequential filtering (Zubeldia-Plazaola et al., 2015).

**TABLE 2:** Enzymes suitable for dissociating cells from solid tumours.

**Enzyme**	**Cancer**	**Derivation**	**Method**	**References**
Collagenase	Cervical	Biopsy	1 mL solution of 0.2% type I collagenase, incubation for 1 h	Villa et al., 2018
	Colorectal	Resection	5–10 mL of collagenase type H solution of concentration 1.5 mg/mL and incubation for 4–6 h or overnight at 37°C	Failli et al., 2009
	Renal	Resection	5 mL of collagenase solution and incubation for 45 min–1 h	Yap et al., 2019
	Breast	Biopsy	0.2 mg/mL collagenase III solution, incubation overnight	Faridi et al., 2018
	Liver	Resection	Triple digestion with type IV collagenase for 5 min at 37°C	Cheung et al., 2014
Collagenase + hyaluronidase	Breast	Surgery/mastectomy	Slow digestion: 1.6 mg/mL collagenase + 0.14 mg/mL hyaluronidase, incubation overnight; Fast digestion: 2 mg/mL collagenase + 2 mg/mL hyaluronidase, incubation for 4–6 h	Zubeldia-Plazaola et al., 2015
Collagenase + DNase	Breast	Biopsy	2 h in 0.15% collagenase and 0.015% DNase	Meltzer et al., 1991
Collagenase + dispase	Ovarian	Surgery	1 mg/1 mL collagenase/dispase solution, incubation for 2 h	O’Donnell et al., 2014
	Brain	Surgery	0.6 U/mL collagenase/dispase solution, incubation for 30, 60, 120 min, or overnight	Volovitz et al., 2016
Collagenase + DNase + dispase	Pancreatic	Surgery	1 mg/mL collagenase, 4 μg/ml dispase and DNase I 1 mL or 2 mL per 0.1 g tissue, incubation for 1 h	Ehlen et al., 2020
	Skin	Surgery	200 U/mL type I collagenase, 200 U/mL dispase and 70 U/mL DNase solution, incubation for 2 h	Dallaglio et al., 2013
Collagenase + hyaluronidase + trypsin	Breast	Surgery	0.5 × collagenase/hyaluronidase solution, incubation for 16 h, then additional digestion with 0.01% trypsin for 30 min	Weigand et al., 2016
Collagenase + DNase + trypsin	Pancreatic	Surgery	1 mg/mL collagenase XI, 4 μg/ml DNase and trypsin 1 mL per 0.1 g tissue, incubation for 30 min	Ehlen et al., 2020
Collagenase + trypsin	Breast	Mastectomy/biopsy	Incubation in 2.5% crude trypsin for 30 min, followed by 0.15% collagenase solution overnight	Pandrangi et al., 2014
Hyaluronidase	Brain	Surgery	1000 U/mL solution of hyaluronidase, incubation for 30, 60, 120 min, or overnight	Volovitz et al., 2016
Dispase	Prostate	–	Digestion with dispase II solution	Wang et al., 2019
	Ovarian	Surgery	2.4 U/mL dispase II solution, incubation for 30 min	Pribyl et al., 2014

A combination of collagenase and deoxyribonuclease (DNase) is effective in breast and ovarian cancer (Meltzer et al., 1991; O’Donnell et al., 2014). DNase gets rid of genomic DNA from samples dedicated to further analysis with RT-PCR^3^. Collagenase and DNase can also be combined with dispase to isolate primary cells from pancreatic cancer (Ehlen et al., 2020). Dispase, also called neutral protease, is a very stable metalloendopeptidase that is less harmful to cells than trypsin and prevents cell aggregation without cell membrane damage (Alvarez et al., 2006). This enzyme provides delicate detachment of epidermal cells and split-up of the epidermis from the dermis^4^. Different combinations of enzymes (Table 1) may be used to isolate primary cells from brain tumour samples, as shown by Volovitz et al. (2016). Research throughout few enzymes (collagenase, DNase, hyaluronidase, dispase, and papain) and their combinations showed great effectiveness of DNase, collagenase, and hyaluronidase (DCH) mixture, as well as, dispase alone. The most favourable enzyme concentrations were: DNase (5 u/ml), collagenase (0.05%), and hyaluronidase (1000 u/ml). Usage of DCH yielded excellent dissociation quality and excellent viability at comparable concentrations. However, the best results were achieved with the use of dispase. In that case, cell survival and tissue dissociations were the highest and the most satisfying (Volovitz et al., 2016). Isolation of primary cancer cells with dispase is also an effective procedure in prostate, ovarian and cervical cancer (Pribyl et al., 2014; Villa et al., 2018; Wang et al., 2019). The use of dispase enables the effective recovery of viable, fibroblast-free epithelial ovarian cancer cells (EOC). The EOC cultures achieved with dispase disaggregation are highly responsive to genetic manipulation (Pribyl et al., 2014).

#### Chemical Dissociation

The cell membrane contains cations, such as Ca^2+^ and Mg^2+^, that provide integrity for the cell surface and intracellular structural matrix. The use of chemical agents enables removing those ions, which causes the loosening of intercellular bonds in, i.e., epithelial cells. The best chemicals used for sequestration are ethylene diamine tetraacetic acid (EDTA), ethylene glycol tetraacetic acid (EGTA), and tetraphenyl boron complexes with potassium ions (Mitra et al., 2013). To isolate primary hepatocytes, EGTA is a popular method of choice. EGTA is a chelator for calcium ions that efficiently disrupts cell adhesion to the underlying matrix. Moreover, removing Ca^2+^ promotes flushing the blood out of tissue specimen, cramps blood clotting, and disturbs the desmosomes among cells (Bhogal et al., 2011; Horner et al., 2019). EDTA acts similar to EGTA, although it is often combined with trypsin and used during trypsinisation to detach cells from a medium and break cell-to-cell adhesion (Kurashina et al., 2019).

#### Mechanical Disaggregation

Mechanical disaggregation of tumour tissues comprises mincing with a scalpel, filtration through 50–100 μm mesh, homogenisation, vortexing, pipetting, or application of extreme osmolarity stress (Mitra et al., 2013). The most frequently used method for mechanical dissociation is cutting tissue samples with scissors or scalpel to the size of 1–2 mm^3^ (Phuc et al., 2010; Ali et al., 2017; Faridi et al., 2018; Ghaderi et al., 2019). Specimens prepared this way can be subsequently placed in a Petri dish and cultured with an explant method, or mechanical disaggregation may be combined with an enzymatic process for better results (Nushtaeva et al., 2019).

Alternatively, instruments like disposable disaggregator or microfluidic device can be used for mechanical dissociation of tissue samples. Semiautomatic tissue disaggregation operates at a speed of 100 rpm, which enables tissue disintegration without trauma. It contains two elements: boreholes surrounded by six microblade, and the other is a rotating element that brings the tissue onto blades (Failli et al., 2009). Microfluidic device technology aims to improve mechanical dissociation of tissue, reduce the time of the procedure, enhance cell recovery, and increase sample purity. The device provides rapid and gentle dissociation with little or no enzyme usage. A microfluidic instrument consists of a network of branching channels that are gradually decreasing in width. The channels have repeated expansions and constrictions that cause a fluidic blow-out, providing the shear force necessary to separate cells (Qiu et al., 2015, 2018).

### Methods of Primary Cancer Cell Culture

Cell culture is a commonly used tool that enables understanding of cell biology, tissue morphology, protein production, and diseases mechanisms. Moreover, they are essential during preclinical research of drugs, including cancer treatment, and conducting studies on gene function. The selection of appropriate cell culture methods may impact finding new treatment strategies or optimising existing radiotherapy and chemotherapy (Kapałczyńska et al., 2016). The cultures may be accomplished under adherent conditions when cells grow as a monolayer, attached to a glass or plastic dish, or maybe cultivated in suspension, which better reflects the natural environment. Up to this point, 2D cultures were the most commonly used type of cell culture, however, 3D cultures are displacing them more often (Figure 2; Jensen and Teng, 2020).

#### 2D Cultures

Cells in adherent 2D culture grow as a monolayer in a particular culture flask or on a Petri dish attached to a plastic surface. The main reasons standing by 2D cultures’ popularity are their low-cost maintenance, fast time of culture formation, and simplicity. Despite that, 2D cultures face many difficulties. One of the disadvantages of adherent culture is its inability to mimic the natural structure of the tumour and express cell-cell and cell-extracellular environment interactions essential during cancer research. In 2D culture conditions, cells lose their diverse phenotype, thus morphology is changed what affects their functions and cell signalling. Moreover, cells in adherent culture have constant access to nutrients, oxygen, and metabolites present in the medium. In the case of cancer cells *in vivo*, nutrient availability is variable due to the tumour’s natural architecture. Lastly, adherent culture is most commonly monoculture, enabling research of one cell type, resulting in the absence of tumour niches (Kapałczyńska et al., 2016).

#### 3D Culture

Since the 1970s in the 20th century, many researchers have contributed to developing 3D cell culture, so cancer studies can be conducted on this model (Penderecka et al., 2020). The 3D culture shows significant improvements in studies concerning cell morphology, proliferation, response to stimuli, and drug metabolism. These advances are possible by a remarkable feature of 3D cultures, enabling observation of cells in their *in vivo* conditions while being cultured *in vitro.* Consequently, 3D cell culture accurately mimics the cells’ environment, preserves natural cell shape and growth. Cells cultivated in this model often keep cell junctions that allow for cell-to-cell communication (Jensen and Teng, 2020).

Moreover, proper cell–cell and cell-extracellular environment interactions, provide arising of environmental niches. Molecular mechanisms, such as gene expression, splicing, topology, and biochemistry of cells, are well preserved by contrast to 2D cultures that tend to interfere with gene expression (Kapałczyńska et al., 2016). The most common 3D models used for cancer research are scaffold-based cultures, multicellular spheroids, and matrix-embedded cultures (Penderecka et al., 2020).

Scaffold-based techniques comprise approaches like hydrogel-based support, hydrophilic glass fibre, or organoids. The use of hydrogels enables mimicking of the extracellular matrix (ECM) and free flow of cytokines and growth factors throughout the tissue-like gel. Hydrogels can be natural and synthetic, and natural ones are made with polymers, such as collagen, fibrinogen, Matrigel, gelatin, or alginate. Artificial models usually use synthetic polymers made up of polyethene glycol, polylactic acid, and poly(vinyl acetate). Polymeric scaffolds are replicating the structure of the ECM very accurately, thus they are an essential tool to study cell-to-ECM interactions. Hydrophilic glass fibres are crucial models for designing 3D tumours and tracking cell migration, however, this model is still being explored. Organoids mimic the microenvironment of organs and are very useful for modelling human diseases by pluripotent stem cells derived from patients (Jensen and Teng, 2020).

The multicellular spheroids model includes techniques such as hanging drop microplates, magnetic levitation, and spheroid microplates. The last approach is especially unique due to its ability to grow freely without any scaffold. Spheroid microplates enable the growth of multicellular cultures because of the large volume. Moreover, cell growth has significant differences between cells grown as multicellular spheres and cultured in 2D culture. The cells from 3D culture showed multidrug resistance, displayed stem-like behaviours, and their motility was increased. The hanging drop method uses gravitation force to induce self-aggregation of cells that further make up spheroids. This approach offers high replicability of results. Magnetic levitation is performed by injecting magnetic nanoparticles into cells that induce cell aggregation to spheroids when exposed to magnetic force. This generates a concentrated environment where ECM can be synthesised, and further biochemical assays may be performed, i.e., western blotting (Jensen and Teng, 2020).

## Methods for Cell Line Characterisation

Cell lines’ characterisation is crucial to identifying cell line origin, purity, and features. The process of characterisation and authentication should include different assays related to specific (extra- and intracellular) markers. The researchers should confirm whether obtained cell lines meet their expectations because, during multiple passages, the cell line may lose its key characteristic features (Geraghty et al., 2014). Additionally, cell line characterisation helps to avoid common misidentification and contamination with mycoplasma. These problems are often ignored by the research community, which leads to modification of scientific data. Studies using misidentified or contaminated cell lines are not reproducible, and their results are uncertain and less valuable. Moreover, such research is not cost-effective (Geraghty et al., 2014; Almeida et al., 2016).

### Immunohistochemistry

Immunohistochemistry (IHC) is a leading method for cancer diagnosis in clinical pathology. IHC uses monoclonal and polyclonal antibodies to identify specific antigens in specimens (Jensen et al., 2017). There are four formats of specimens used in IHC: paraffin-embedded, frozen, free-floating, and cytological. Out of these formats, the most commonly used is the paraffin-embedded approach. The tissue sample is fixed in formalin to preserve proteins *in vivo* state as much as possible, thus fixation is the most significant step. Then the sample is processed in paraffin wax, which provides a sort of supporting media. After that, tissue fragments of 4 μm thickness are dissected and placed in a water bath at the temperature of 45°C. The specimen is laid on a microscope slide and dried at 37°C overnight for better adherence of tissue to the slide. The sample is rewaxed before immunohistochemical staining (Renshaw, 2017). IHC is one of the most widespread tools for assessing cells’ features such as expression of hormonal receptors (ER and PR), EGFR receptor (HER2 neu) in breast cancer (Bonacho et al., 2020). IHC may also be used to mark epithelial cell characteristics in ovarian cancer by applying specific antibodies (EpCAM, CA125, and MOC-31) (O’Donnell et al., 2014).

### Western Blot

Western blot (WB) is a commonly used method to separate and identify proteins. Initially, a mixture of proteins is separated during electrophoresis. Then, the proteins are transferred to the membrane, which is incubated with specific antibodies. Incubation is followed by washing which causes a rinse of unbounded antibodies. Antibodies bounded to proteins are detected and visualised on the membrane. The thickness of each band depends on the amount of protein (Mahmood and Yang, 2012). WB is useful while examining the expression of biochemical markers. For instance, prostate epithelial cells express various marker proteins during differentiation, thus their activity can be investigated by WB. It enables the characterisation of the molecular subtype of cell lines, including ones expressing CK18, CK 5, E-cadherin, or p63 (Wang et al., 2019). In ascending colon cancer, WB can be useful for the determination of the presence and size of insulinoma-associated protein 1 (INSM-1) from lysates. INSM-1 is a product of gene *INSM-1* that regulates the expression of neuroendocrine markers (Shinji et al., 2019).

### Flow Cytometry

Flow cytometry (FC) is a prominent method used in immunology, molecular biology, and cancer research. This approach utilises single or multiple laser light to analyse cells and particles suspended in buffered solution. Each particle undergoes analysis under visible light scatter and fluorescent assessment. Visible light scatters evaluated particles in the forward direction, which displays the comparative cell size and at 90°, which shows the cell’s complexity and granularity. Fluorescent assessment includes cell transfection and fluorescent proteins’ expression, staining with fluorescent dyes, or fluorescently conjugated antibodies. Visible light scatters independent of fluorescence measurement. The most common purpose of FC is immunophenotyping. It enables analysis of cell culture for various parameters, such as T and B cell markers, monocyte markers (CD14 and CD11b), NK cell markers (CD56 and CD161), activation markers (CD69 and CD25), memory markers (CD45RO and CD27), cytokines presence (IFN-γ, TNF-α, and IL-2 define TH1 cells), and antigen-specific markers (MHC). Cell line’s features can be analysed in terms of proliferation (markers Ki67 and CFSE) and apoptosis (the activity of caspases) (McKinnon, 2018). Like other methods, FC helps categorise cell lines according to the presence or absence of particular cell surface markers defining the origin of isolated cells (epithelial, mesenchymal, or stem cells) (Widowati et al., 2018). Moreover, FC enables the determination of the cell line’s molecular subtype by analysing overexpression of receptors, such as HER2 or HER3 in breast cancer (Nushtaeva et al., 2019). FC serves as a straightforward tool for quick evaluation of stemness markers like aldehyde-dehydrogenase (ALDH) (Tusa et al., 2020). Moreover, FC is useful not only for immunophenotyping, it has an application assessment of cell apoptosis, viability, and cell cycle, for example in gastric cancer (Zhao et al., 2018).

### Immunocytochemistry and Immunofluorescence

Immunocytochemistry (ICC) utilises labelling cells with antibodies to determine population homogeneity and molecular profile. This method visualises individual cells and enables evaluating the distribution of molecular markers in the cell culture. Immunocytochemistry includes cell morphology and exposes subcellular localisation of a particular antigen. The experiment should consist of the negative control (without primary antibody) and positive control (with a cell known to express given antigen) to determine staining propriety. Immunocytochemistry often uses fluorescently tagged secondary antibodies to reveal the primary antibody bounded to an epitope on the investigating molecule. A secondary antibody can detect the heavy chain of the primary antibody. This procedure utilises prolonged incubation for the primary antibody, followed by a few washes to remove the remaining antibodies. Then shorter incubation is applied for a secondary antibody with subsequent washing. Afterwards, the sample is processed under microscopy (Ziaeian et al., 2012). The immunocytochemical staining may unveil the cell lines’ features, such as no ER, PR, or HER2 expression in breast cancer cell lines (Ghaderi et al., 2019). ICC is also useful to detect keratins’ presence, for example, keratin 19 a biomarker for many tumours, including breast cancer (Shi et al., 2017; Sharma et al., 2019).

Immunofluorescence (IF) is a method of choice while working with adherent cell lines. It simplifies the isolation of the individual signal and cell lines usually are not vulnerable to damages, due to similar auto-fluorescence as they exhibit from tissue sections (Renshaw, 2017). The immunofluorescence is also a useful approach to reveal cytoskeleton structures like filaments, i.e., vimentin and cytokeratins (Lai et al., 2015), as in the case of pancreatic cancer (Ehlen et al., 2020). Immunofluorescence is often used to detect mycoplasma contamination by mycoplasma-specific polyclonal or monoclonal antibodies (Freshney, 2010).

### Confocal Fluorescent Microscope

A confocal fluorescence microscope serves as a great tool for assessments in cells and tissues. Confocal microscopes generate high-contrast images through optical sectioning. It is achieved by a high-resolution objective lens which produces optical sections thinner than 1 μm without slicing the specimen. A single confocal image, also called a slice, is enough for quantification, although it is possible to use a series of confocal images to generate a 3D dataset. The 3D model enables the reconstruction and quantification of the whole sample volume. The use of fluorescent molecules is advantageous while assessing molecules’ localisation from various cellular compartments (Jonkman et al., 2020). In practice, the confocal fluorescent microscope can be used to distinguish morphological differences, for example, between pericytes and embryonic fibroblasts. After fixation, cells are labelled with fluorescent dyes, and immunostained cells are imaged on the confocal microscope (Zhao et al., 2019).

### RT-qPCR

Gene expression measurement in cultured cells by reverse transcription-quantitative real-time polymerase chain reaction (RT-qPCR) is an essential technique used in molecular biology and medical research (Van Peer et al., 2012). This tool provides the measurement of RNA levels through cDNA in a qPCR assay and monitors the amplification of targeted DNA samples by fluorescence of dyes used in the reaction. Fluorescence is equivalent to the amount of product yielded during the PCR cycling phase (Fowotade, 2018; Adams, 2020). Model systems can be subjected to inhibitors, stimulants, small interfering RNAs, and knockouts to investigate gene expression. Moreover, RT-qPCR is commonly used as quality control to examine changes in expression during RNA sequencing (RNA-seq) (Adams, 2020). In the characterisation of cell lines, RT-qPCR can be used to investigate cancer stem cell markers, such as expression of clusters of differentiation (CD24, CD44, CD133, and CD166) (Shinji et al., 2019). This method also enables the detection of particular genes needed for cell line characterisation, for example, B2M and RPL29, in the case of tongue carcinoma cell line (Wang et al., 2017). RT-qPCR can be applied for the determination of epithelial-mesenchymal transition (EMT), which contributes to the migration property of cells. For this assessment, synthesis of primers for EMT (E-cadherin, N-cadherin, and Vimentin) and CSC markers (CD44v6, CD117, ALDH1A1, and Snail) is crucial (Deng et al., 2019).

### Karyotyping

Karyotype analysis includes pairing and ordering all organisms’ chromosomes, resulting in the genome-wide projection of the individual chromosomes. The preparation of karyotypes consists of staining, enabling exposure to chromosomes’ structural characteristics (Raghavendra and Pullaiah, 2018). Karyotypes are often used to investigate the aberrations in chromosome numbers, chromosomal translocations, deletions, and inversions (Sampson and McGuire, 2014). For this reason, karyotyping is a cheap and popular method of choice to demonstrate changes in the cell line and reveals exciting features of cell lines, that have never been observed before (Geraghty et al., 2014; Wang et al., 2019).

### Next-Generation Sequencing

There is an increasing demand for high throughput technologies for gene expression analysis of cell lines (Van Peer et al., 2012). In this instance, NGS provides comprehensive cell line characterisation taking into account transcriptome, proteome, metabolome, and genome. The most useful methods are whole-genome sequencing and whole-exome sequencing to describe cell lines’ genomes. Transcriptome characterisation may be provided by assays using RNA, such as RNA-seq and MicroRNA sequencing. Cell lines’ proteome is most commonly assessed by chromatin profiling and protein quantification (RPPA), whereas, metabolite profiling is used to describe the metabolome (Ghandi et al., 2019). Predictive markers for drug resistance, biomarkers for diagnostic purposes, and prognostic markers in lung cancer have been discovered and analysed by quantitative proteomic analysis (Hoi et al., 2017). Transcriptome analysis of breast cancer stem cells revealed pathways activated in CSCs, in particular, eIF2, eIF4, and ephrin. Transcriptome examination of breast CSCs also uncovered differential methylated DNA, histone modifications, and shed a light on tumour suppressor genes downregulated in CSCs (Li et al., 2018).

## Conclusion

Primary cell lines are pivotal for cancer research and have significantly contributed to understanding tumour biology, molecular processes, oncogene activation, and gene expression of individual patients. Primary cancer cultures are crucial for conceptualising therapeutic targets that may serve for future drug development or personalised cancer therapy. Patient-derived samples are currently extensively used for the improvement of existing treatments in personalised medicine. Moreover, the combination of primary cancer cell lines with advanced techniques (such as NGS) is a prospective cancer research tool that leaves the door open to identify novel biomarkers and oncogenes. Knowing the limitations and benefits of primary cell lines, scientists should pay more attention to the choice of model they will work on. It is important to also use established cell lines according to the purpose of the planned experiment. Nevertheless, the culture of primary cell lines is a key element in research towards personalised medicine, which is the future of cancer treatment.

## Author Contributions

MR and OP wrote the manuscript with support from MM, IP, WMS, and TT. MR and OP declare an equal co-authorship. MM and IP participate in drafting the article and helped supervised the article. WMS and TT make substantial contributions to the conception and design of the article and give final approval of the version to be submitted and any revised version.

## Conflict of Interest

The authors declare that the research was conducted in the absence of any commercial or financial relationships that could be construed as a potential conflict of interest.

## Footnotes

1 Regulation (EU) No 536/2014 of the European Parliament and of the Council of 16 April 2014 on clinical trials on medicinal products for human use, and repealing Directive 2001/20/EC. https://eur-lex.europa.eu/legalcontent/EN/TXT/?uri=celex%3A32014R0536.

2
http://www.worthington-biochem.com/CLS/default.html

3
http://www.worthington-biochem.com/DNASE/default.html

4
http://www.worthington-biochem.com/DISP/default.html
